# Care of the Deciduous Teeth

**Published:** 1908-02-15

**Authors:** O. W. White


### CARE OF THE DECIDUOUS TEETH.

BY O. W. WHITE, D.D.S.

[Read before the Detroit Dental Society, Nov. 14, 1907.

In presenting this subject for discussion this evening, I was told to cover the subject very generally, as this is the only evening of the year devoted to this part of dentistry.

As volumes have been written on this subject, good, bad and indifferent, and many more will be written in the future before we are able to accomplish what dentistry should, for this period in the development of the oral cavity, you can see what an impossible task I have before me in the short time at my disposal to properly review any one of the three divisions into which I have divided this subject, namely:

1. Oral Prophylaxis—Its practice in the care of the deciduous teeth.

2. Operative Dentistry—Selection of material for restoration of lost tooth structure.

3. Orthodontia—Its application in prevention of malocclusion and aid in proper development of the maxillae

I shall only attempt to call your attention to the most important points of what should be done for our little patients during this period, and leave the subject of detail in the different operations to be brought out in the discussions.

Dentistry neglected the deciduous teeth for a great many years, because of the fact that little importance was attached to the part they played in the future of the oral cavity.

The object of dentistry being restoration to normal function of impaired organs, the dentist considering when this was accomplished his duties ceased, the same mistake has been made by the medical profession

To-day the object in dentistry is to prevent harmful influences interfering with normal functions, and maintenance of the highest degree of vitality possible to all tissue of the oral cavity.

With this object in view it must be plain to all that the greatest attention, energy and skill should be exercised on the developing tissues of the child, the same as upon plant and animal life, if we expect an ideal condition when maturity is reached.

Before going into the importance of oral prophylaxis, I wish to call your attention to two of the most common causes of neglect of this branch of dentistry. The first is the difficulty experienced by many in performing the required operations on the little patients. This, I think in a large per cent of the cases is the failure of the operator to appreciate the mental condition that children are suffering from when brought to the dentist for the first visit. From listening to experiences of their brothers and sisters, and even their fathers and mothers on their return from visits to the family dentist, they have good reasons to be in a nervous state of mind when visiting the office for their initiation into this work, and the utmost care should be exercised to win the child's confidence, and until you are successful in this work, I think it is a great mistake to attempt any treatment whatever, even if it is necessary for the child to visit you a number of times before you succeed in allaying his fears, and he can feel confident that the least possible pain will be inflicted upon him. You will surely be repaid for your trouble in future operations.

Unless a man's nature is such that he loves children, he has made a great mistake in choosing dentistry for a profession, and he will never be able to attain the success possible to the man who has a natural sympathy, love and admiration for his young patients.

The second reason why the deciduous teeth have not received the proper attention is that the compensation for services rendered to them has not been in proportion to that received from operations on the permanent teeth. The censure for this condition should be put upon our predecessors, as they taught the public to believe that little attention was necessary to the first teeth, and when these troubled the patient they were removed at half price, or free of charge, for no other reason I suppose than because it required so little muscular exertion to perform the operation, they did not feel that they deserved reward for this service, and at this day we know that their consciences directed them rightly.

Time spent in educating patients as to the proper method of caring for deciduous teeth and in performing dental operations upon such teeth requires an expenditure of energy skill and intelligence for which the dentist should receive a just compensation, and it is his duty to demand it.

Oral Prophylaxis—its field in the care of the deciduous teeth is a large one, and of the most vital importance to the developing mouth.

The use of the term "Ora prophylaxis" is criticised by some as being improperly used, that may be true to some extent, yet the common use of this term in dentistry to-day has given it a position that custom wi l be the authority for its use. Its interpretation by the dental profession to-day is the prevention and cure of all harmful influences upon the teeth, and the raising of the tissues of the oral cavity to the highest state of vitality possible. There is no doubt, from the success attained by its practice, that oral prophylaxis has established a permanent position, and is essential to the practice of dentistry.

Dentistry will have attained its highest ambition of success only when it has accomplished the preservation of all tissues of the oral cavity, from youth through old age, in their normal healthy condition, performing all functions nature demands of them, thus aiding the development ot the body of the child, and in sustaining the body in after years.

I believe that oral prophylaxis is going to accomplish more to this end than any treatment advocated heretoiore.

Its method of practice and the results obtained have been demonstrated to this Society on several occasions. It will not be necessary for me to explain them, but I wish to emphasize the most important feature without which your results will only be failures. It is thoroughness in the minutest detail. This coupled with perseverance, is sure to bring great satisfaction to all who attempt its practice.

It would be folly for me to attempt to enter in any detail of the part operative dentistry exerts on the preservation of the deciduous teeth. I will leave that for the discussion. My practice being limited to orthodontia, it has not permitted me to keep in touch with this line of work.

The materials that have been most successful in the preservation of the deciduous teeth, and which seem more adapted to these conditions, are oxyphosphate of copper, gutta percha and amalgam, the use of porcelain and gold being limited.

The oxyphosphate of copper as it is now furnished to the dental profession in its new form, is certainly a vast improvement over the old product, and I think it will be more extensively used in the future

The objection to color being offset by ease of manipulation and its germicidal properties, a dense permanent filling may be obtained with proper care and mixing. This material seems to have an embalming effect on the tooth structure, which may be noticed in specimens of teeth filled with this material that have been extracted. Wholly decalcified dentine beneath the filling will have been changed to a horny mass, and semi-decalcified dentine will be more dense than normal dentine in another part of the tooth. Even when the embalmed tissues have become thoroughly bare of filling, it resists caries producing agents to a very marked degree.

The adhesive property of oxyphosphate of copper makes it very useful in cavities of deciduous teeth, this property of adhesion being much greater than in the oxyphosphate of zinc. Should the pulp be injured or destroyed, it is a good plan to fill root canals, pulp chamber and cavity with this copper filling, providing tooth has only a short time to rem ain in the mouth. On account of its embalming effect on the tooth structure, it would not be advisable to resort to this procedure when absorption has not taken place to a marked degree. There is no better root canal filling where teeth are required to remain in arch for any great length of time than gutta percha. Gutta percha, amalgam, porcelain and gold are all familiar to you, and it will not be unnecessary to speak on them.

Great care must be exercised in preserving proper contour and should spacing be lost, no filling should be inserted till the proper separation has been obtained so the tooth can occupy proper posit'on in the developing arch.

We may give the greatest attention to oral prophylaxis and operative procedures, and yet fail in our duty to our patients if we neglect the most essential factor or function the deciduous teeth have to perform. The influence on the development of arches and occlusion of the permanent teeth. The deciduous teeth are seldom in malocclusion when erupted, but certain influences develop during the life of these teeth to cause this condition.

What complications and perplexing problems of the permanent teeth could have been avoided had they been diagnosed and received the proper treatment when the deciduous teeth were present, for at this time the arches are easily corrected on account of the small amount of mineral deposits in bone structure, and large amount of connective and elastic tissues. It is the moral duty of each and every one to see that our young patients when malocclusion exists, to properly inform them as to the condition and possibilities of early treatment, whether you undertake its correction or not proper diagnosis should be made of these cases at the earliest possible age.

I will enumerate some of the causes which are responsible for malocclusion, showing slides and models to better illustrate these conditions. It is very difficult to say how much we can hold hereditary condition responsible for malocclusion of deciduous teeth, but I am inclined to think it is very little at this time of developing of the arches.

The essayist here showed by lantern slides between 35 and 40 illustrations of cases where malocclusion has occurred of the most troublesome kind, because of lack of attention to the erupting permanent teeth, and the care of the deciduous teeth until they shall have passed the critical periods.

I wish to say, in the majority of slides of cases thrown on the screen, a proper diagnosis could have been made at the ages of 3 to 5 years, and proper preventive treatment applied, so that the natural process of development would not have been interfered with. The orthodontist should not be called upon to cause an absorption and reconstruction of the tissues, but he could have aided nature at the proper time so the tissues would develop normally. By simple means growth may be stimulated at this age which in later years may be very difficult.

A common mistake made in diagnosis of young patients by the dental profession in regard to the first observation of malocclusion, is the belief that nature can correct the arrest of development. It will only in such cases where the cusps of permanent teeth can develop normal occlusion, or near enough, so the incline planes can guide them to proper position. Otherwise, every month delayed is only mak- the case more complicated.

In conclusion, I wish to bring before the Society for discussion this evening this subject in which I have been much interested, and one we must sooner or later realize as a duty of our profession.

America has been in the past a leader in the profession of dentistry, yet to-day I think one of the greatest movements for advancing this work and giving the public the greatest benefit and knowledge, must be credited to a foreign country. That is the teaching and enforcing of ora hygiene in schools. Are we to wait for commercial enterprises to take up th s work for the purpose of financial gain, at the expense of professional dignity? I think not. Very few present who have given this subject a thought, but can see we would make a great mistake by doing so.

DISCUSSION.

Dr. Homer E. Parshall. Mr. President and Ladies and Gentlemen: Dr. White's subject of this evening I consider to be one of the most important up for discussion before the Detroit Dental Society this year; and I say this not that I do not thoroughly appreciate the importance of the remainder of the programme. Dr. White's paper is timely, and I hope he has convinced you all of the necessity of treating these little patients thoroughly and conscientiously when they come into your hands. By doing this you will gain the love and the confidence of these children, which will follow you for years, and be of far greater worth to you than the dollars for which we all so often expend our energies. My favorite patients are these little ones, and the littler they are ordinarily, the better I like them. The brightest spot in a day spent at my office is when some little tot runs up to me and puts its arms around my neck.

As Dr. White says, first we must gain the confidence of these little ones, and you can only do that through honesty. The success of early oral prophylaxis depends much upon the mothers, and we should never grow weary in instructing them in regard to their duties in this respect. Tell them time and time again, if necessary. If the mother does her duty and the dentist does his duty, the child has a good start with 32 sound teeth in its second childhood.

Early oral prophylaxis is a great thing all along the line from childhood to childhood, and, as Dr. White has pointed out to you, the all important thing in oral prophylaxis is thoroughness.

In regard to the restoration of tooth structure, we know that many cases of mal-position are due to improper fillings. We have all seen these large amalgam fillings on occlusal surfaces if it is an inclined plane. We notice this more especially, however, in the first molar. This inclined plane has a tendency to disturb the normal mesiol-distal relation of the first molar, tending to throw the case into a full-fledged second or third-class malformation Then there are the crowned cavities which are filled too full, and which may have a similar effect in permitting the opposing cuspid to sink into its normal position; but the most important of all is the contouring of the deciduous teeth. You seldom see properly contoured durable fillings in the deciduous teeth. The necks of the deciduous teeth are much smaller in proportion to the crown than in the permanent teeth, and by not contouring the fillings properly they are liable to crowd together, permitting the first molar to crowd forward, resulting of malformation in the second and third class.

There are many other very perplexing questions arising in regard to the treatment of the deciduous teeth. I think decidedly more if we treat them conscientoiusly than in the permanent teeth. We have conditions running all the way from nearly exposed pulps to the alveolar abscess.

Going back to the filling material for the approximal cavities, I have always preferred amalgam fillings. The Oxyphosphate of Copper cement I have not used sufficiently to form a definite opinion. I hope that it will prove all that Dr. White has claimed for it. I have been using it some, and am, so far, pleased with the results. I would not, however, fill the roots of the temporary teeth with gutta percha. The pulp chamber can be filled with gutta percha or the other materia'.

I am very glad that Dr. White was able to show us so many very beautiful slides this evening, as they will assist us in forming our diagnosis, and we should all be able to diagnose developing cases of malformation in the deciduous teeth. The slides have beautifully shown the effects of premature extraction of the deciduous teeth, and when such authorities as Cryer, and the American Text-Book of Operative Dentistry, says that when deciduous teeth are preventing the eruption of permanent teeth into their normal positions, they should be extracted: there is no wonder that the profession at large is often guilty of performing this harmful operation.

Dr. M. T. Watson. Dr. White politely invited me to keep out of this, so I will be very brief.

The only thing that occurred to me that I would care to speak about, in view of the discussion that is coming on after this, is the recommendation that Dr. White made in regard to the use of oxyphosphate of copper. In former years when I was doing a regular practice, I used that considerably, and it has many virtues; but I would urge you to be very cautious how you fill cavities on the mesial or distal surfaces of the deciduous molars or the cuspids with any material which greatly wastes away, owing to the action of the secretions of the mouth. There it is necessary to put a durable filling that will permanently retain its form and prevent the teeth from closing together, as they will when cement filling dissolves away. I know that a filling between the deciduous molars for instance, that is made of oxyphosphate of copper cement disappears very much slower than the ordinary oxyphosphate cements, still it does waste away, and as that material wastes away, the teeth keep crowding together. The first permanent molar erupting behind the deciduous molars causes the entire deciduous arch to move forward, and it is by that process that the lengthening of the jaws takes place. But with a cement filling in this place, instead of the whole jaw being pushed forward, the second molar is simply pushed further towards the second deciduous molar, and consequently contraction of the arch is almost sure to take place. That same condition holds true in regard to cement fillings in the permanent teeth. I have seen many, many cases where a marked injury to the occlusion had taken place from no other cause in the world except the wasting away of cement in cavities between the teeth. While I know from my own experience that men are often tempted to use cement because of the greater ease in placing it where teeth are sensitive, etc., yet it is really a very great injustice to use cement in proximal cavities, and leave it there any great length of time. If you want to use it temporarily, well and good; but take it out and replace with a permanent filling before any injury takes place, because after it takes place it is extremely difficult to get enough separation to restore the mesiol-distal diameter of that tooth, and, if you do get it, you may get it by drifting the teeth in the wrong direction, which disturbs further the distal relations.

Dr. E. B. Spalding. I would like to ask Dr. Watson if I should be criticized for the practice I have of filling between deciduous molars with gutta percha, where the cavities were not large enough to put in metallic fillings and contour them? And also for another reason, where possibly the patient was of such a nervous disposition that he would not allow a proper preparation of the cavity, to pack hard gutta percha between them and link the two cavities together. I should like to know if this is a procedure that would be cr ticized. It certainly preserved the space, and also produced more space, and possibly that would be the criticism; but there would be more space than is necessary or would be right.

Dr. Geo. Burke. I was particularly interested in the feature of the paper which referred to the examination of school children by competent dentists. I have read a number of articles in the various dental journa s on this subject, and it is something that of course every progressive man in the profession is very anxious to see brought about. As we have with us this evening an ex-member of the board of Education of Wyandotte, I should like to hear from him as to what the possibilities would be here in this city for taking up this work in our schools. Dr. Hall, it seems to me, ought to enlighten us on this subject.

Dr. J. S. Hall. I think it was very emphatically stated at one of the meetings here that I came from Delray. Feeling rather proud of that, I rather resent the implied compliment that I came from Wyandotte. Now I do not feel that I can greatly enlighten you in regard to what could be done in this matter. While I was a member of the Board of Education we had so many things to do in the way of advancing education in our district, and the conditions were so different from what they might be in the city here, that we considered ourselves compelled to look after other things that we deemed more important, but had we succeeded in remaining out of your city corporation, we might have perfected our educational system until we reached the dental hygienic curriculum. Consequently, I have not given the matter any particular thought. I can see that there is a great field for it, but I hardly think that the time is ripe yet, because I think that the educational system of the City of Detroit possibly needs a little cleaning up in other particulars before it can take on even such a needed improvement. I may be wrong, but I think there is a good deal of house-cleaning to be done here, and I hope the people of Detroit would be well satisfied if we could get the educational system made over from what it is. I think when that is done, we will be in a posit on to look after the teeth of the children.

Dr. G. C. Bowles. I think this paper is very timely, and I would like to see further facts brought out and emphasized. We talk here about educating the parents. I had occasion recently to rea ize that the dental profession also needs educating. An incident happened to me within the last few weeks that po nts this moral. A father brought a six-year-old child to my office whose teeth were in a very much neglected condition. Nothing had ever been done because the parents regarded the teeth as but temporary, and soon to be lost, the tooth-ache would last perhaps a year or so, but that did not matter, they would be replaced by other teeth, and it was not worth while doing anything. The father brought the child in, and I told him that it was necessary that the teeth shou'd be saved, if possib'e. One temporary first molar had a pulp nearly exposed. I filled the other teeth, with some assurance that the fillings would hold and the teeth be comfortable, but I said that this tooth may ache, but I do not want to remove the pulp. So I capped it and filled t. A few days afterwards the tooth ached, and the father was very much provoked. He did not tell me so, but I heard it from a neighbor. He called at my home one evening when I happened to be out, so he went to a dentist in the vicinity, who told the father that the man who had filled this tooth was a grafter, and that there was no sense in filling temporary teeth. When a dentist can make such a statement as that, it seems as if education should begin nearer home. Young men who ought to know better are telling patients that these teeth will last but a little while, and nothing should be done. How often we see these six-year molars destroyed. Often the fault is with the dent st, patients have been to the dentist frequently, and have not been told. They say often, "Why didn't my dentist tell me that it was a permanent tooth." I think such facts need the most pronounced emphasis at our dental Society meetings.

With reference to fillings: I have used the oxyphosphate of copper now for four or five years, and I find on the whole, that it gives me more satisfaction than any other one material that I can use. Most of us, I expect, know that we ought to preserve the normal space in the deciduous teeth, but when you get molar teeth in children with approximal cavities, with the pulp nearly exposed, it is not the easiest thing to restore that contour with amalgam fillings. If you get sufficient undercuts in these teeth to sustain that filling you will very likely have tooth-ache. Of course, amalgam is the ideal filling, but it is sometimes next to impossible to to insert. The only thing we can use in many cases with any degree of satisfaction is something like the oxyphosphate of copper, and in my experience it will answer where nothing else would. I have been in the habit of doing as Dr. Spalding has done, where there were approximal cavities, using gutta percha, and I think it is a very satisfactory procedure.

With reference to the examination of children's teeth in schools, Drs. White and Watson and a few others discussed that subject thoroughly two years ago. We believed at that time, and still believe, that the time is ripe for the discussion of this thing, and its introduction in the public schools. The school system already is overcrowded, and the children have more than they can do with their studies, and to learn what they have to learn, but if the teachers themselves were instructed, the kindergarten teachers for instance, they could instruct their little pupils at that early age in the need of the thorough use of the tooth brush, and at that time a great deal could be done toward inculcating habits that would go with the children, and help to preserve the teeth. Oftentimes a word from the school teacher means more than a dozen or a hundred from the parents at home, and a word or two could be dropped without adding any great burden to the teacher or the children, if the teachers were instructed to give that word, and I believe they would do it if they were required to.

Dr. Zederbaum has shown that this thing can be handled in the schools, and that the schools are not only ready to take it, but receive it eagerly. He has done a great deal at a sacrifice of time and money, and much credit is due him. He has gone about throughout this county and made examinations of the children's teeth, and finds that the teeth of the school children in this state are as bad, if not worse than in any other part of the world, according to statistics gathered by men who are competent to do the work. He states that if the dentists in this county had nothing to do but fill the teeth of these children that he examined in the schools, that it would take them a whole year—all of them for a whole year filling the cavities that at present exist in the mouths of the school children. Something ought to be done, it seems to me, in the way of instructing our school teachers. Dropping a wise word where it will be of most good in the early days of school life, is wisdom indeed.

I think this subject might well be taken up again this year perhaps, with the idea of securing the co-operation of the school authorities.

Dr. M. T. Watson. It just occurred to me that this little suggestion may be of value. We often find cavities in approximal surfaces of deciduous teeth broken down to such a degree that it seems impracticable to attempt to put in permanent fillings, and I have seen very little attempt made in the way of anything else in that class of teeth, but it is possible in cavities of that sort to cement a piece of platinum wire into the filling so that it comes into absolute contact into the approximal surface of the adjoining tooth, it will be retained after a greater part of the cement has washed away, and when it has washed away so that it produces an unhygienic condition, simply repair the cement filling and leave the platinum wire still cemented in place. Where it is impracticable to make extensive fillings, you will be surprised at what you can do by simply imbedding this platinum wire, letting the end of it come in contact with the approximal surface of the adjoining tooth.

Dr. W. A. Giffen. I have thought a great deal about the subject of teaching oral hygiene in the schools, and when I was president of this Society three years ago a committee was appointed to look into the matter. They thought that it might be a good idea to consult with the Superintendent of Public Schools of Detroit, and make some arrangement, if possible, to have literature distributed among the school children, giving them the needed information. The committee had several meetings, worked hard, were received courteously, and that was all.

Now, according to my ideas, I think it is up to the Society to do some work. We must show the school authorities not only how this work should be done, but we must show them the necessity for doing it. We can show them that Dr. Zederbaum, in his county, found that 75 or 85 per cent, whatever it was, of children's teeth were affected with caries or other troubles, but that don't do any good here.

It does not make any difference if a committee of ten or twenty told our Board of Education that such a condition exists here if we cannot show it. If we have some charts made, and get enough members of this Society to co-operate, and examine the children in the lower grades of the schools, collecting data showing the exact conditions, then we should have a basis to work on, and by doing that I think we could demonstrate to their satisfaction our position, and we could then demand something. Now if we go and make this representation to the Board of Education while, as Dr. Ward says, they are swamped with various fads they wish to introduce or improve in the schools, and the general opinion I think to-day is that we have too many books and too much for the pupils to learn, the authorities will surely say we are doing it from a selfish standpoint. We have got to show the necessity for having this work done, and it is up to this Society to do it. In order to find out what we can do, I think we should take a vote and find out how many would volunteer to give a certain amount of time each week to such work. I think if we could get 30 or 40 members of the Society to put in a few hours each week, and make a record of the conditions found, I think it could be done. It seems to me that this is the only way to get at it.

DR. GEORGE BURKE. In this connection I read an article in the *Digest*, I think it was for September, and it recited a case in Richmond, Va., where the dentists appointed a committee to interview the school board, and they were not successful; so one of the committee, a Dr. Fisk by name, decided that with the support of the members of the profession in that city he would run for School Inspector; and after his election he succeeded in inducing the school bord. to have examinations made. Dr. White tells me that they have accomplished the same thing in New York City. I have seen the book that is used in New York City, Dr. V. C. Dell's book, entitled "Popular Essays on the Care of the Teeth and Mouth." It is illustrated and gotten up in a very attractive manner, and there is a small time spent each week in some of the grades studying this subject. I should like to see, some time in the future, some member of this Society run from his ward for School Inspector. I think it would be preferable to have him elected or delegated by the Sociey to run, and I would suggest further that this Society bear some tof the expense in connection with con ducting his campaign. After he is elected he could send on to New York and get a few hundred copies of Dr. Dell's book and proceed to talk the Board up to the importance of this subject. This might not appeal to a great many as practical, but this work of a committee does not seem to be effective, so far as I can find out. You have got to elect one of your number to get in there. Committees in Baltimore, San Francisco and Chicago, I believe, waited on the School Boards with absolutely no results.

Dr. J. S. Hall. If Dr. Burke will take the trouble ot investigate the school system of the city of Detroit, and learn how the election of an inspector to the school board is carried on, he will find that this Society is not rich enough to contribute even partially to the expense of the election of our man. I venture to say that I could win a bet that there is not a single man here who can go out and run as School Inspector, as a dentist, and be elected. You have got to take into consideration the fact that we have a peculiar system of politics here in Detroit, and there is not money enough in this Society to elect a man to that board. If Dr. Burke has the money to spend I would like to see him run, and I would do all I can to support a man like Dr. Burke, for he would be a strong improvement on some of the members we have today. When you want scientific politics, our school board is where you will find it. There is no use in our sending a delegate down to the school board to see about this, because they will not pay any attention to it. If you can succeed in getting a man on the school board in this city we might see a ray of light in the future; but we shall have to increase our dues to more than $2 a year.

Dr. Hoff. It seems to me that Dr. Bowles struck the keynote of the situation when he said that it is not the school boards that we need to educate, neither is it the parents of the children that we need to educate, but it is the dentists themselves. We have got to come home with this whole subject before we can attempt to do anything in the way of teaching others. You cannot teach until you realize the facts yourself. Apparently most dentists do not believe that caries of the teeth is a preventable disease. If they do believe it they do not practice what they believe. Dr. Bogue, of New York City, three or four years ago read a paper at Atlantic City on the subject of preventing dental caries. He made the statement there that dental caries was the most preventable disease of any that generally attacks the human body. In a letter that I recently had from him he reiterated this statement. He asked me if I was of the same opinion, and he wrote me emphatically that he was. Dr. D. D. Smith has made a statement of very similar import to that which Dr. Bogue has made. He claims that dental caries is a preventable disease, and that we should not have it at all. You all know what Dr. Smith's remedy is for this. Dr. Bogue's methods are not quite the same as Dr. Smith's. His methods are to work with children very much along the lines which Dr. White has attempted tonight to lead us; that is, by correcting mal-positions of the teeth. He advocates the beginning of the correction of these mal-positions of the teeth of the children with the deciduous teeth, and continuing close supervision of the teeth throughout all their course, until such time as the permanent teeth are completely erupted. In this letter to which I refer, he said that he had a number of patients that have grown up under his care from childhood. He has had entire supervision of their teeth during that time, and they have never lost a deciduous tooth or permanent tooth because of dental caries—all because he took these children in the beginning, and saw to it that their teeth erupted in their proper position, and then he was careful to maintain these teeth in their integrity until the time came for them to be lost physiologically. He has been practicing this throughout all his career, and has found that as long as he could control his patients, he has never lost a tooth. Now every dentist cannot control his patients in this way; he will say, therefore, this is not practicable. I have many times felt that if I were a younger man I would gather about me half a dozen children, at least, and I would get the parents to make an agreement with me that I should have absolute control of these children's teeth, and I would undertake to carry them through the whole period of the development of their teeth until the permanent teeth had erupted, and demonstrate whether it was possible to develop a permanent set of teeth of full integrity. There are, of course, accidents that might interfere with the success of such a research, but I firmly believe from the experience I have had, that it is possible, and that Dr Smith and Dr. Bogue are both right in their contention that it is not only possible, but practicable to preserve all the teeth of our children until they are fully developed and grown in a perfectly intact position. This accomplished the future would be easy. How can we do this? Only by getting absolute control of these children. You say, it is not practicable. It is practicable, and it is certainly ideal; and we ought to work for it more consistently than we do. I dare say there are many here that have such ideals; but many have no other ideal than to meet conditions as they present themselves. We do not take enough interest in the teeth of children as they are presented to us from day to day to enforce any proper ideals. In that respect it seems to me that *we* need educating. We need to have our ideals raised to that point where we wil' make some sacrifice and effort, and will strive to work out such a grand ideal. If some one would do this with a few patients and demonstrate it to our satisfaction, it would make it possible and almost necessary that we should carry out this treatment with every patient that comes into our care. It seems to me that even with the patients as they now come to us, if we would correct the malocclusions or preserve the deciduous teeth in proper form, with proper filling material, we shall learn the way to ultimate success. I believe the ideal filling for deciduous teeth is none of the materials that have been mentioned to-night, but should be one that will restore the entire tooth in its normal form and detail, even to the restoration of the cusps of the teeth, so as to secure absolute occlusion in the natural order. This may seem impracticable, but I believe it is a coming possibility, in view of the fact that we are now developing a system of cast inlay fillings, with which it wil be possible to save every deciduous tooth in its full form, so as to make it occlude with its opposing teeth in such a way that it will give us the proper development of the bones of the jaw in which the teeth are located. It is only by some means whereby we can restore these teeth to their proper occlusion, so that the children will masitcate their food properly that they will develop the jaws and face symetrically in the proper physiological way. Such teeth will also be kept clean by a normal and proper mastication Now, if we could only get the dentists to appreciate the possibilities and practicability of doing such things as this, we shall not have to spend much money on school boards to get them to take up this matter seriously. Some one will then take care of the poor children. There are to-day plenty of people who could afford to have such work done that now do not have it done simply because we have not believed in our ability to get results that will justify us in asking the public for adequate compensation for our services. When we know that we can produce results that are ideal, and can conserve children's teeth and produce healthful conditions in their mouths and bodies, it will be no trouble to get people to spend the money necessary to secure such results. They will come without trouble, and you will find the women will be willing to sacrifice sealskin coats, and men will go without automobiles in order that their children may have perfect teeth and healthy bodies. It remains with us to have sufficient faith in ourselves that we can convince our patients that such things can be done, and few will be found so poor as to be unwilling to give up money if they or their children can have perfect bodies.

Dr. R. E. Bailey. I am very much interested in the subject, and that old saying that "charity should begin at home" certainly applies here, when we say that we should educate ourselves. I had a case the other day where a second lower deciduous tooth had been prematurely extracted, and the first permanent molar stood about on an angle of 45 degrees, mesially and lingually. The dentist who extracted the tooth never raised an objection to extracting it; even said that the deciduous teeth should not be filled at any time. We have several practitioners in and about our city, who tell their patients, as the dentist Dr. Bowles cited did, that it is not the proper thing to fill the deciduous teeth at any time. For that very reason I wish I had them all down here to hear this discussion.

Dr. J. F. Spring. (Pontiac). I hear these same things nearly every day in my own office, and recently had a patient from Dr. Bowles' office with the complaint that he would not extract his teeth. I have learned some good things from the paper of Dr. White to-night—some things that have made me somewhat sad, because I suppose such things happen only out in the country where I live. I am glad I came here to-night, as I have received a new impulse. I came down to find out what was to be done about this re-organization work, and hope that we can have our men from Pontiac, who are all good fellows, join with you in the grand work. I should like to see them all here to-night, and hope they may become interested in this work you have had under discussion.

Dr. E. T. Loeffler. In talking so much about politics and the examination of children's teeth and ideals, we are wandering somewhat from the practical consideration of care of the deciduous teeth.

In considering the deciduous teeth, it seems to me that we cannot eliminate the first permanent molar. I like to divide deciduous teeth into two periods: that preceding the eruption of the first permanent molar, and that following its complete eruption. The handling of little children is quite a perplexing problem, and when you take into consideration the fact that their teeth are generally neglected, and note that there are not such a great number of very bad malocclusions because of it, it seems to me that the premature loss of some deciduous teeth is not of such great importance as some seem to think. I have been studying children for a quite time, and I think habit has more to do with malocclusions than the loss of these teeth. During the time preceding the eruption of the first permanent molar we should try and preserve all the temporary teeth. The first permanent molar erupts posterior to all the temporary molars, and when it is normal in mesio-distal relation and fully erupted, it firmly holds the jaws at the right distance from one another, so that all other incoming permanent teeth can erupt to the proper length, and in that way is determined the bite and its length. It also marks the boundary line between the deciduous teeth and the permanent molar teeth posterior to it; that is, it prevents the incoming permanent bicuspids from erupting too far backward, and the second and third molars from erupting too far forward. During the period following the eruption of the first molar and the anterior teeth, the deciduous teeth are not so firm in their sockets as before. They begin to loosen and they are shed. The occlusion is not good, and function depends mainly on that first molar which is permanent. We have an entirely different state of affairs when the first permanent molar is not and when it is in place.

Now, much has been said about oral prophylaxis of the deciduous teeth; but I have yet to record the first little patient who has had the skill, and the mother who has been willing to give the time and the patience to thoroughly care for that little childs' teeth. I think in many instances it would require the father and all the rest of the fami'y to succeed. You might have to convert the baby's old high chair into a dental chair. Stop to consider all the neglect, and then see what reasonably good results people have in the way of fairly good teeth.

As to the question of extraction, one hesitates to approach that subject, because when you speak of the extraction some will say emphatically, never extract. Years ago many believed in extracting the first molars, sometimes whether they were decayed or not. There is a time when you absolutely must extract, having weighed and considered everything, and the question is, when is that time. I know where some children have been blessed by the extraction of certain teeth. Years ago many first molars were extracted, and some good results followed. Supposing a case where the first molar is all broken down with decay and you try to restore it, but it will not keep quiet; you cannot do much for it. Should we extract a tooth like that? It may be built up, and again it breaks down, and we have the expense of a crown, which we find will not hold. Shall we extract such a tooth at the age of seven, or at the age of eight or nine years? If first molar preserves the length of the bite and if you extract it too soon, your lower jaw will come closely to your upper and you will have a large overlap. The best time to extract this tooth is when the second permanent molar and first permanent bicuspid are in occlusion, or when their occlusial inclines are coming properly. That will be at about the age of eleven.

I like ideals, but if we stick to them too constantly we cannot get practical results. I believe in the spurring of a man to do the practical and the very best. There are many things to consider in taking care of deciduous teeth, and I like to wait until we have the complete eruption of the first molar, and then look out for bad habits of the individuals.

Dr. O. W. White. In replying to Dr. Spalding's question as to gutta percha fillings, I would say they are ideal in some cases, especially when they do not have to remain long, and even when they do they can be watched and restored. If it is making too much space, change it; of course a little too much is better than not enough, but where you can control your patient where you can see it, often it is a very nice way to take care of approximal cavities where you are unable to put in a more permanent material; but, of course, the ideal is some more permanent filling.

In response to the remarks of Dr. Loeffler in regard to the first molars holding the deciduous molars in position, I fear he will often meet disappointment, as so strongly attached a tooth as the first molar will move. To claim that the six-year molar will stand up in the arch, and you can remove the deciduous molars, and that six-year molar will stay in the normal position is absurd, for it will not. It certainly will drift forward; in not one case out of a thousand would it remain in the normal distal relation in the arch.

In saying that these cases are very perplexing in regard to retaining the proper spacing, etc., I would say that it is not one-quarter as perplexing as it is to correct malocclusion in the permanent teeth later on in the life to the patient. While it is difficult and hard for you to spend the time and hard work with the patient, and when you consider that time is responsible for a great many of the bad conditions of malocclusion later in life, you will realize what a great benefit you will do your patient by correcting all the careous conditions, and retain good interproximal spacing If it is necessary to extract, and there are lots of cases where you have to, by all means retain the proper spaces in the arch. It is very little trouble to make retainers for spaces in arches where you have to extract, and the benefit you will obtain, if you have that patient for a number of years it will save you hours of work later on. In all your dental operations it will aid you, if you have no malocclusion in any of the teeth.

In regard to the other subject, that of school hygiene, Dr. Burke said I spoke of New York as having hygiene in the schools. I had letters from about eight cities. There is not one that has a regular examination of the pupils in this country, that I know of. Dr. Burke has spoken of Richmond. Of course, I do not know anything about the conditions there. New York City voted last year on laying aside a certain amount for compulsory examinations of the mouths of the pupils in schools, and it was defeated. The dentists there hope that there will be a certain amount appropriated next year for this purpose. There are no regular examinations of pupil in Boston, Chicago, Toronto, Pittsburg, and other cities.

"In Germany compulsory education has been given to the care of the teeth and mouth. In a report of the city of Strasburg, there were 12,691 visits to the dental clinic in 1904, and 6,282 teeth were treated. Of 2,269 children between the ages of three and four years examined, only 362 had sound teeth; less than 16 per cent of 2,103 children between the ages of six to eight examined, only 160 had sound teeth."

"Since the introduction of the treatment there is a marked improvement in the general health of these public schools, and there is less headache and stomach trouble."

Two years ago the Superintendent of our schools said that it would take a good many years before any special appropriation could be made for this work. He said that there was a mothers' meeting, where the parents gathered together with their children, and that they would be willing to extend us an invitation to give talks to these mothers at their regular meetings, but of course all expense would have to be borne by our Society. What the present condition is I do not know. I do not know whether they would accord the privilege to this Society now or not.

